# FAM225A facilitates colorectal cancer progression by sponging miR‐613 to regulate NOTCH3

**DOI:** 10.1002/cam4.3053

**Published:** 2020-04-28

**Authors:** Xuexiu Zhang, Haoling Shi, Jianning Yao, Yanle Li, Bing Gao, Yanzhen Zhang, Chunfeng Wang, Haining Zhou, Lianfeng Zhang

**Affiliations:** ^1^ Department of Gastroenterology The First Affiliated Hospital of Zhengzhou University Zhengzhou China; ^2^ Department of General Surgery The First People Hospital of Zhengzhou Zhengzhou China

**Keywords:** colorectal cancer, FAM225A, miR‐613, NOTCH3

## Abstract

Colorectal cancer (CRC) is a fatal disease ranking the third among the commonplace cancer types around the world. It is extremely significant to exploit effective treatments against CRC. FAM225A was proved to influence cell progression and forecast unfavorable prognosis in nasopharyngeal carcinoma. The role and function mechanism of FAM225A are still unclear in CRC. In this research, FAM225A was discovered presenting much higher expression in CRC tissues and cell lines. In addition, depleting FAM225A was capable of inhibiting cell proliferation, migration, and epithelial‐to‐mesenchymal transition (EMT) progress, and enhancing cell apoptosis ability. Furthermore, miR‐613 exerted important effects as a mediator between FAM225A and NOTCH3. NOTCH3 was negatively correlated with miR‐613, whereas was positively associated with FAM225A. Via competitively binding with miR‐613, FAM225A positively regulated NOTCH3 expression. FAM225A facilitated CRC occurrence and development through positively regulating NOTCH3 expression by binding with miR‐613. In a word, FAM225A/miR‐613/NOTCH3 axis may play a tumor‐facilitator in CRC cell progression. These data manifested the pivotal effect of FAM225A/miR‐613/NOTCH3 pathway in CRC cell proliferation, apoptosis, and migration process. The findings may provide some theoretical basis and different perspective for CRC treatment.

## Graphical abstract

FAM225A presents high expression in CRC tissues and cell lines. FAM225A expedites CRC cell proliferation, migration abilities, and impairs cell apoptosis capacity. FAM225A acts as a sponge of miR‐613 and is negatively correlated with miR‐613.

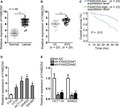

## 1 INTRODUCTION

Colorectal cancer (CRC) is a fatal disease ranking third among the commonplace cancer types around the world.^1^ According to statistics, there are approximately over 500 thousand patients dying of CRC per year worldwide.^2^, ^3^, ^4^ In China, CRC is the fourth most usual malignant tumor with increasing morbidity and mortality.^4^, ^5^ Like many other cancers, CRC development is a multistage progress, including the accumulation of genetic and epigenetic alterations.^6^ The symptoms of CRC patients are influenced by multiple factors, like different stages of tumor, tumor location, and size.^7^ Although diagnosis and treatment for CRC have been improved, the 5‐year survival rate of CRC remains relatively low, and effective strategies for preventing CRC recurrence are in great need. Owing to the unfavorable prognosis of CRC, it is extremely significant to exploit new treatments against CRC.

Long noncoding RNAs (lncRNAs) have become new hot spots for disease treatment, especially for cancers.^8^, ^9^ Recently, multiple researches revealed that lncRNAs exerted important effects during genesis, development, and progression of human tumors, as well as served as the driving factors of carcinogenic function via many mechanisms.^10^, ^11^, ^12^, ^13^ Particularly, some lncRNAs were associated with cell fate and gene expression in the development and progression of several cancers, including CRC. Though lncRNAs do not engage in protein coding process, they are capable of regulating gene expression at epigenetics, transcription, and other levels. LncRNAs are closely correlated with genome modification, transcriptional activation, and transcriptional interference.^14^ Plenty of studies have proved that the aberrant expression of lncRNAs is closely connected with cell growth, proliferation, migration, and invasion.^15^ For example, as a tumor promoter, upregulated LINK‐A predicted unfavorable prognosis for metastatic non‐small cell lung cancer.^16^ LncRNA FAM225A was proved to enhance cell proliferation, migration, invasion, and tumor growth in nasopharyngeal carcinoma (NPC). Working as a competing endogenous RNA (ceRNA), FAM225A could upregulate ITGB3 via binding with miR‐590‐3p in NPC.^17^ However, the role and function mechanism of FAM225A are still unclear in CRC.

This research aimed to study how FAM225A affected cell functional behaviors in CRC. The ceRNA role of FAM225A helped us probe the molecular mechanism of FAM225A in CRC cells. Interestingly, miR‐613/NOTCH3 axis was found being regulated by FAM225A, and played a significant part in the progression of CRC. This paper may offer new perspectives for searching biomarkers for CRC treatment.

## 2 MATERIALS AND METHODS

### 2.1 Tissue samples

About 46 CRC tissues and adjacent normal tissues were gathered from patients who were diagnosed at the First Affiliate Hospital, School of Medicine, Shantou University from May 2013 to June 201. Participants did not receive any treatment before surgical resection and signed written informed consent. All tissue samples were frozen in liquid nitrogen and stored at −80°C. This study was approved by the Ethics committee of the First Affiliate Hospital, School of Medicine, Shantou University.

### 2.2 Cell culture

Normal human colorectal mucosal cell (FHC) and CRC cells (HT29, HCT116, SW620, and SW480) were bought from Chinese Academy of Sciences. Cells were cultured in RPMI‐1640 medium (Invitrogen) containing 10% fetal bovine serum (FBS; Invitrogen), then mixed with 1% penicillin/streptomycin (Sigma‐Aldrich) and cultivated in 5% CO_2_ incubator at 37°C.

### 2.3 Cell transfection

HCT116 and SW620 cells were transfected with specific shRNAs against FAM225A (sh‐FAM225A#1#2) and the negative control (shNC), and pcDNA3.1/FAM225A, pcDNA3.1/NOTCH3, and the empty pcDNA3.1 vector (all from GenePharma), respectively. The miR‐613 mimics and NC mimics were synthesized by GenePharma. Each plasmid was transfected into cells using Lipofectamine 2000 (Invitrogen).

### 2.4 Quantitative real‐time polymerase chain reaction (qRT‐PCR)

Total RNA of cells was isolated utilizing Trizol reagent (Invitrogen). Total RNA was reversely transcibed into cDNA using Reverse Transcription Kit (QIAGEN, Frankfurt, Germany). The ABI PRISM 7000 Fluorescent Quantitative PCR System (Applied Biosystems) was employed to progress the qRT‐PCR. The relative expression was calculated with the application of 2^−∆∆Ct^ method. GAPDH or U6 was used as internal reference.

### 2.5 CCK‐8 assay

A cell counting kit‐8 (CCK‐8, Dojindo, Japan) was used to evaluate cell proliferation. Transfected cells were cultured in 96‐well plates (1000 cells/well) for 0, 24, 48, and 72 hours at 37°C. Next, each well was added with 10 µL of CCK‐8 solvent. Then, cells were cultured for another 4 hours. A microplate reader at 450 nm (SYNERGY4, USA) was applied for measuring the optical density.

### 2.6 Colony formation assay

HCT116 and SW620 cells were transfected and cultivated into 6‐well plates for 2 weeks. The culture medium was replaced every 3 days. The colonies were fixed and dyed by methanol (Solarbio) and crystal violet (Sigma‐Aldrich). Finally, colonies were counted through a microscope (Olympus).

### 2.7 EdU incorporation assay

EdU experiment was conducted with Cell‐Light EdU DNA Cell Proliferation Kit (RiboBio, Guangzhou, China). 3 × 10^4^ cells were plated into 24‐well plates and incubated for 48 hours. After incubation in 300‐µL EdU for 2 hoours, HCT116 and SW620 cells were fixed by 4% paraformaldehyde (Solarbio) and stained by Apollo Dye Solution. DAPI was applied to dye nucleic acid. Images were collected via an inverted fluorescence microscope (Olympus) and proportion of EdU‐positive cells was examined.

### 2.8 Western blot

RIPA lysis buffer adding protease inhibitors was employed for extracting total protein. Proteins were isolated by the utilization of SDS‐PAGE, and then moved to polyvinylidene fluoride membrane. After being sealed with nonfat milk, proteins were cultured with primary antibodies for E‐cadherin (ab194982), N‐cadherin (ab202030), Vimentin (ab193555), MMP2 (ab37150), MMP7 (ab205525), MMP9 (ab38898), Bax (ab32503), Bcl‐2 (ab185002), Caspase 3 (ab13847), Cleaved caspase 3 (ab2302), NOTCH3 (ab23426), and GAPDH (ab8245) from Abcam (Cambridge, USA). Then, secondary antibodies were added for cultivating for 1 hour. The amount of proteins was evaluated by chemiluminescence detection system.

### 2.9 JC‐1 assay

Cells were cultivated with 10‐mmol/L JC‐1 (Beyotime) for 30 minutes. The fluorescence‐labeled cells were rinsed in phosphate buffer saline (Solarbio) and analyzed through an EnSpire Reader. The fluorescence rate at 590‐nm vs 530‐nm emission was used to measure the mitochondrial membrane potential. The data and images were analyzed using GraphPad Prism 5 statistical software (Graph‐Pad Software, Inc).

### 2.10 Transwell Assay

The migration abilities of CRC cells were determined by transwell chambers. 2 × 10^4^ cells were added to the upper compartment supplied with serum‐free medium, while the bottom chamber was filled with 10% FBS medium. After being incubated for 48 hours, we fixed cells by paraformaldehyde followed by dying them in crystal violet. Five randomly chosen fields under a microscope (Olympus) were applied to count the number of migrated cells.

### 2.11 Subcellular fractionation

The separation of cytoplasmic and nuclear RNA in HCT116 and SW620 cells was conducted using Cytoplasmic and Nuclear RNA Purification Kit (Norgen Biotek Corporation, Thorold, ON, Canada). Then the subcellular fractions were examined using qRT‐PCR. U6 (nuclear control) and cytoplasm (cytoplasmic control) were employed for normalization.

### 2.12 Fluorescence in situ hybridization (FISH) analysis

RNA FISH probe for FAM225A was produced by RiboBio. Nuclei were counterstained with DAPI. A laser scanning confocal microscope (ZEISS) was used to observe cells.

### 2.13 Luciferase reporter assay

The wild‐type (Wt) and mutant (Mut) binding sites of miR‐613 in FAM225A sequence or NOTCH3 3′‐UTR were subcloned into pmirGLO dual‐luciferase vector (Promega) to construct FAM225A‐Wt/Mut and NOTCH3‐Wt/Mut, and then co‐transfected with indicated transfection plasmids into HCT116 and SW620 cells. The luciferase reporter vectors luc‐miR‐613‐Wt/Mut were established utilizing the interacting sequences of miR‐613 in 3′‐UTR of the seven possible mRNAs. Their luciferase activity was detected by Dual‐Luciferase Reporter Assay System (Promega).

### 2.14 RNA immunoprecipitation (RIP) assay

EZ‐Magna RNA‐binding protein immunoprecipitation kit (Millipore) was applied for conducting this experiment. HCT116 and SW620 cells were lysed by lysis buffer. Then cell lysates were incubated with the RIP buffer containing magnetic beads conjugated with human anti‐Ago2 antibodies (Millipore) or anti‐IgG (Millipore). IgG was seen as negative control. Finally, the RNA was isolated and then analyzed by qRT‐PCR to examine the expression levels of FAM225A, miR‐613, and NOTCH3 in the precipitates.

### 2.15 Statistical analysis

Statistical analysis was processed using SPSS 13.0 (SPSS Inc) and data were shown as mean ± SD. Significance of the variance in groups was evaluated by Student's *t* test or one‐way or two‐way ANOVA, with *P* < .05 as threshold. Gene correlation analysis was performed by Pearson's correlation analysis and Kaplan‐Meier analysis was used for overall survival. Experiments were conducted for at least three times.

## 3 RESULTS

### 3.1 FAM225A presents high expression in CRC tissues and cell lines

**FIGURE 1:**
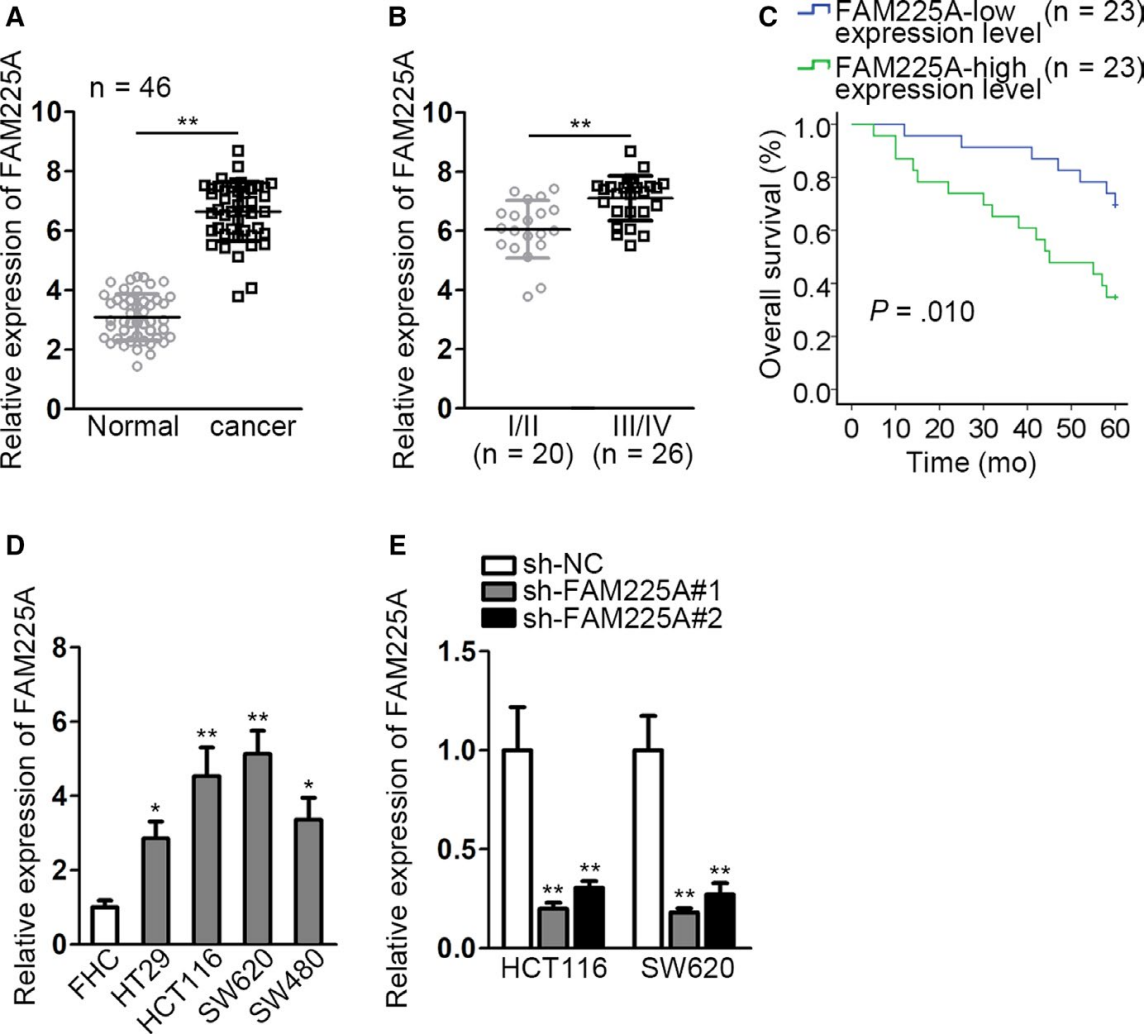
FAM225A presents high expression in CRC tissues and cell lines. A, qRT‐PCR analysis of FAM225A expression in cancer tissues and normal tissues. B, FAM225A expression in different stages of CRC was detected by qRT‐PCR. The PCR pattern used in (A and B) was real‐time PCR, with the PCR results analyzed via 2^−∆∆Ct^ method. C. qRT‐PCR was conducted to determine FAM225A expression in normal cell (FHC) and CRC cell lines (HT29, HCT116, SW620, and SW480). D. Kaplan‐Meier analysis of CRC survival ratio of patients with high or low FAM225A expression. E. qRT‐PCR analysis of FAM225A expression under the condition of interfering FAM225A. Results were presented as the mean ± SD. *P < .1, **P < .01

In the first place, we assessed FAM225A expression in tumor tissues and normal tissues by qRT‐PCR. The higher expression of FAM225A was discovered in tumor tissues than normal tissues (Figure 1A). Besides, as Figure 1B reflected, FAM225A expression in advanced stages (III/IV) of CRC patients was much higher than that in early stages (I/II) of CRC patients. In addition, from the result of Kaplan‐Meier analysis, CRC patients with high FAM225A expression bore lower survival ratio than patients with low FAM225A expression (Figure 1C). In the same time, qRT‐PCR was also utilized to observe FAM225A expression in normal cell (FHC) and CRC cell lines (HT29, HCT116, SW620, and SW480). We found that FAM225A showed much lower expression in normal colon epithelial cell line FHC than in CRC cell lines (Figure 1D). Due to the relative higher expression of FAM225A in HCT116 and SW620 cells, we chose HCT116 and SW620 cells for further investigations. More interestingly, we reduced FAM225A expression in HCT116 and SW620 cells by constructing plasmids containing sh‐FAM225A#1 and sh‐FAM225A#2. As predicted, FAM225A expression was obviously downregulated by FAM225A interference (Figure 1E). In summary, FAM225A harbored high expression in CRC tissues and cell lines, and it was correlated with unfavorable prognosis of CRC.

### 3.2 FAM225A expedites CRC cell proliferation, migration abilities, and impairs cell apoptosis capacity

**FIGURE 2:**
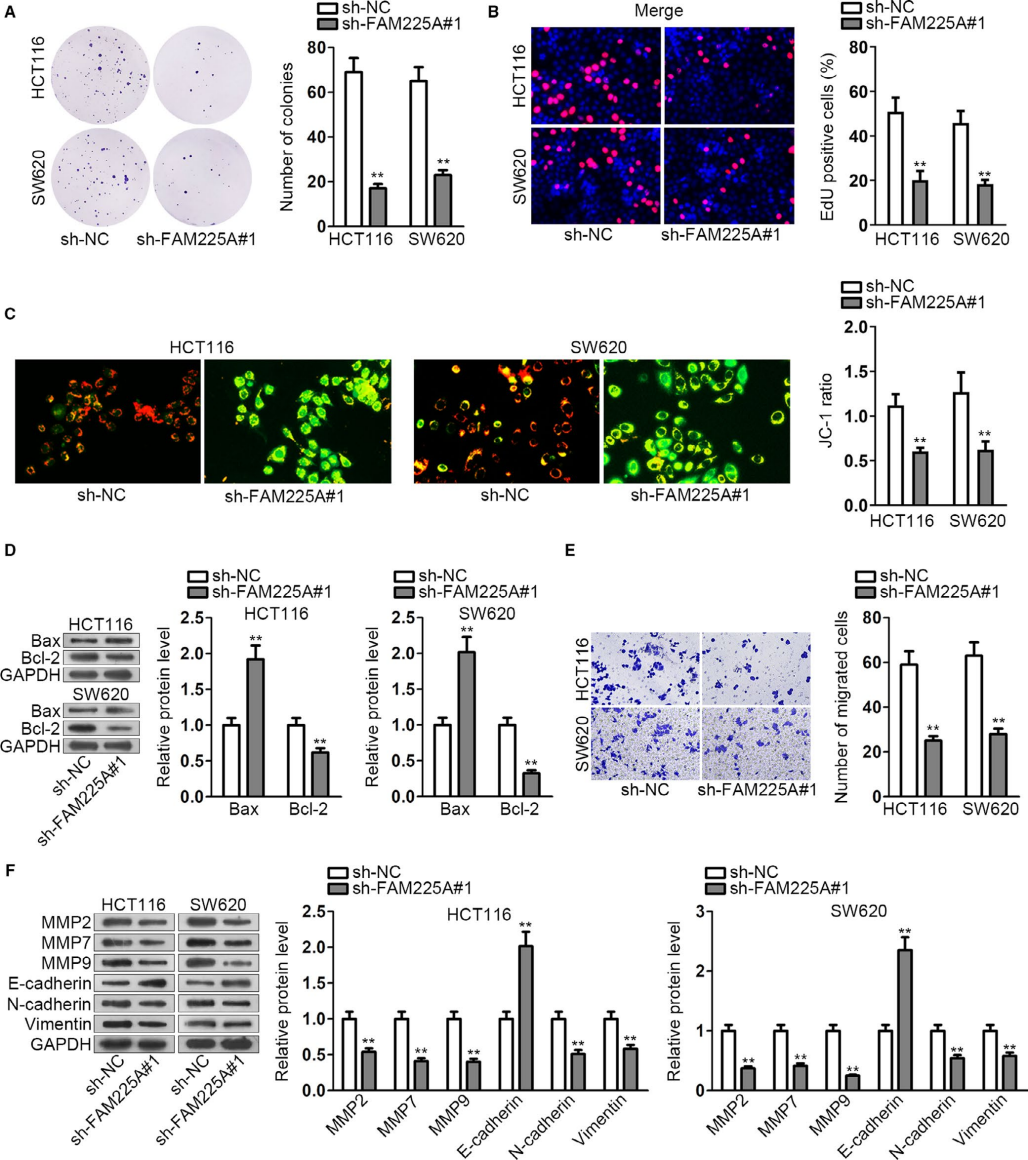
FAM225A expedites CRC cell proliferation, migration abilities, and impairs cell apoptosis capacity. A and B, HCT116 and SW620 cell lines were transfected with the shRNA targeting FAM225A (sh‐FAM225A#1). Colony formation assay and EdU assay were implemented to detect cell proliferation ability. C, JC‐1 assay was performed to assess cell apoptosis capacity. D, Western blot analysis of apoptosis‐related protein levels (Bax and Bcl‐2). E, Transwell assay was performed to assess cell migration capacity. F, Western blot analysis of migration‐related protein levels (MMP2, MMP7, and MMP9) and EMT progression‐associated protein levels (E‐cadherin, N‐cadherin, and Vimentin). All data were displayed as the mean ± SD. **P < .01

Loss‐of‐function assays were carried out to proof the enhancing effects of FAM225A on CRC cell biological behaviors. First of all, FAM225A was downregulated by transfecting sh‐FAM225A#1/2 into HCT116 and SW620 cells. As was in CCK‐8 assay, cell viability was significantly inhibited by silenced FAM225A (Figure S1A). Colony formation assay depicted that the number of colonies was observably declined by downregulated FAM225A (Figure 2A, Figure S1B). EdU assay also manifested the decreased EdU‐positive cells (Figure 2B, Figure S1C). All the assays demonstrated the impaired cell proliferation by FAM225A interference. Meanwhile, cell apoptosis ability was determined by JC‐1 assay and associated protein levels detection. JC‐1 assay showed the descended JC‐1 ratio under the transfection of either sh‐FAM225A#1 or sh‐FAM225A#2 (Figure 2C, Figure S1D). From the western blot analysis of cell apoptosis‐related protein (Bax and Bcl‐2) levels, Bax level was increased, while Bcl‐2 level was declined (Figure 2D, Figure S1E). Also, protein levels of caspase 3 and cleaved caspase 3 were detected. Caspase 3 was not impacted and cleaved caspase 3 protein levels were significantly enhanced by silenced FAM225A (Figure S1F). These assays reflected that cell apoptosis ability was enhanced by interfering FAM225A. Furthermore, the result of transwell assay displayed the restricted cell migration capacity by reducing FAM225A (Figure 2E, Figure S2A). Furthermore, migration‐related proteins (MMP2, MMP7, and MMP9) levels and EMT progress‐associated protein (E‐cadherin and N‐cadherin and Vimentin) levels were examined. Migration‐related protein (MMP2, MMP7, and MMP9) levels were all distinctly decreased. Meanwhile, the expression of E‐cadherin, the epithelial marker, was augmented, while the expressions of N‐cadherin and Vimentin, the mesenchymal markers, were cut down (Figure 2F, Figure S2B). The findings revealed the blocked migration capability and EMT progress owing to FAM225A reduction. Altogether, FAM225A could expedite CRC cell proliferation, migration abilities, and EMT process, while impair cell apoptosis capacity.

### 3.3 FAM225A acts as a sponge of miR‐613 and is negatively correlated with miR‐613

**FIGURE 3:**
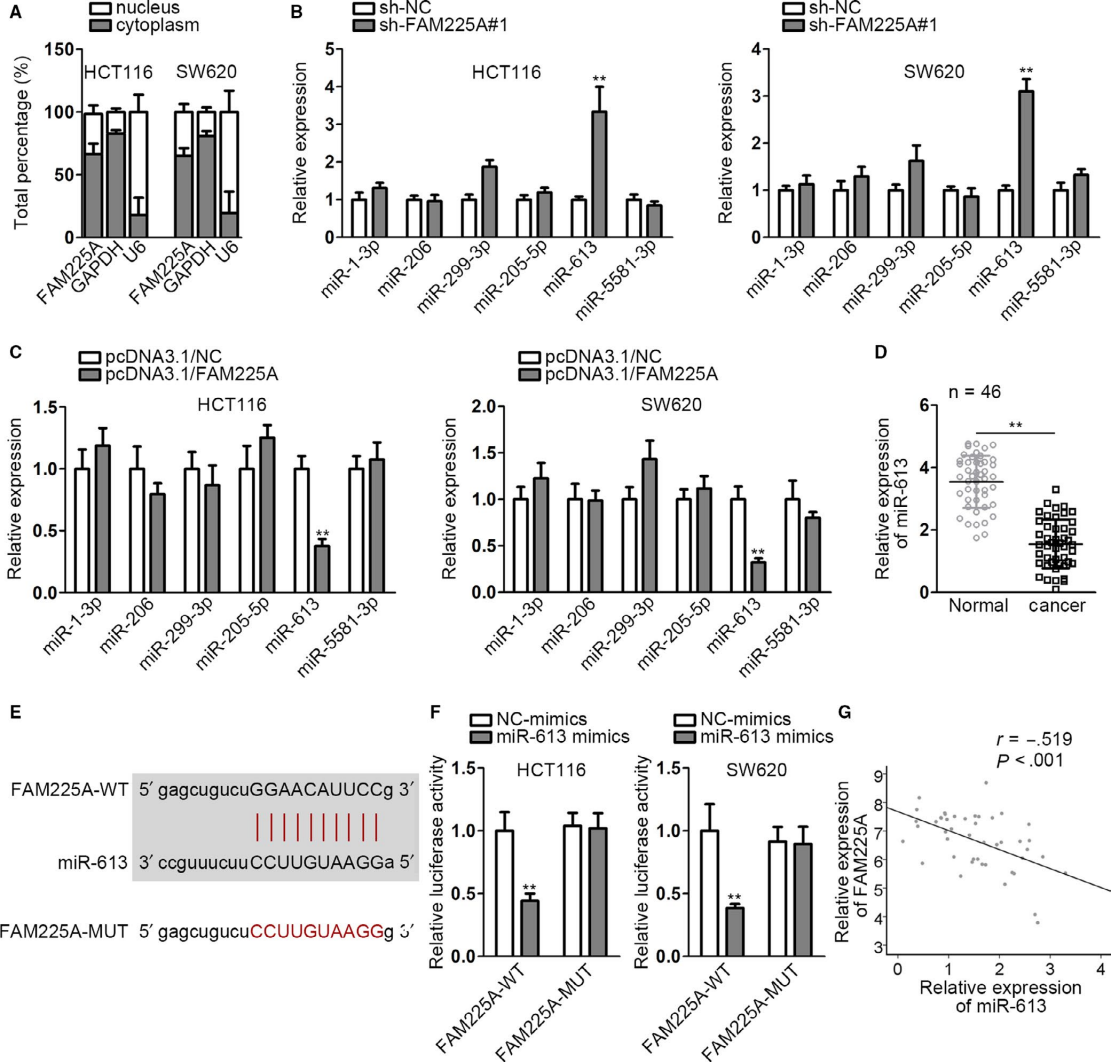
FAM225A acts as a sponge of miR‐613 and is negatively correlated with miR‐613. A, Subcellular fractionation assay was utilized to detect FAM225A location in CRC cells. B and C, qRT‐PCR analysis detected miRNA expressions under the circumstance of interfering or increasing FAM225A. D, qRT‐PCR analysis of miR‐613 expression in cancer tissues and normal tissues. E, The binding sequence of miR‐613 and FAM225A was obtained from starBase website. F, Luciferase reporter assay was to proof the interaction relationship between miR‐613 and FAM225A. G, The inverse correlation of miR‐613 and FAM225A was revealed from Pearson correlation analysis. Results were shown as the mean ± SD. **P < .01, ***P < .001

Subcellular fractionation and FISH assay were adopted to ascertain the location of FAM225A in CRC cells. As shown in the figure, FAM225A was mainly scattered in the cytoplasm of CRC cells (Figure 3A, Figure S2C). Since the ceRNA pattern is a typical posttranscriptional mechanism, we supposed that FAM225A may act as a ceRNA engaging in CRC cell biological functions. We obtained several candidate microRNAs (miRNAs) which may bind to FAM225A from starBase website (http://starbase.sysu.edu.cn). Through interfering FAM225A, we observed that only miR‐613 expression was upregulated (Figure 3B). Simultaneously, FAM225A overexpression only reduced miR‐613 expression (Figure 3C). The result reflected that only miR‐613 could be affected by FAM225A. Thus, miR‐613 was ascertained as the downstream gene of FAM225A. Similarly, we determined miR‐613 expression in tumor tissues and normal tissues via qRT‐PCR. MiR‐613 presented much lower expression in cancer tissues than in normal tissues (Figure 3D). The complementary base pairings of miR‐613 and wild‐type FAM225A (FAM225A‐WT) or mutant‐type FAM225A (FAM225A‐MUT) were acquired from starBase website (Figure 3E). Furthermore, luciferase reporter vectors containing the wild‐type or mutant‐type FAM225A and miR‐613 mimics were co‐transfected into HCT116 and SW620 cell lines. As Figure 3F demonstrated, the luciferase activity of FAM225A‐WT group was remarkably declined, but without any differences in FAM225A‐MUT group. It proofed the interaction relationship between FAM225A and miR‐613. In addition, we measured FAM225A expression under the condition of miR‐613 overexpression. As expected, FAM225A expression was markedly downregulated by miR‐613 increase. Pearson correlation analysis manifested that FAM225A was inversely associated with miR‐613 (Figure 3G). Overall, FAM225A acted as a sponge of miR‐613 and was negatively correlated with miR‐613.

### 3.4 FAM225A modulates NOTCH3 expression by binding to miR‐613

**FIGURE 4:**
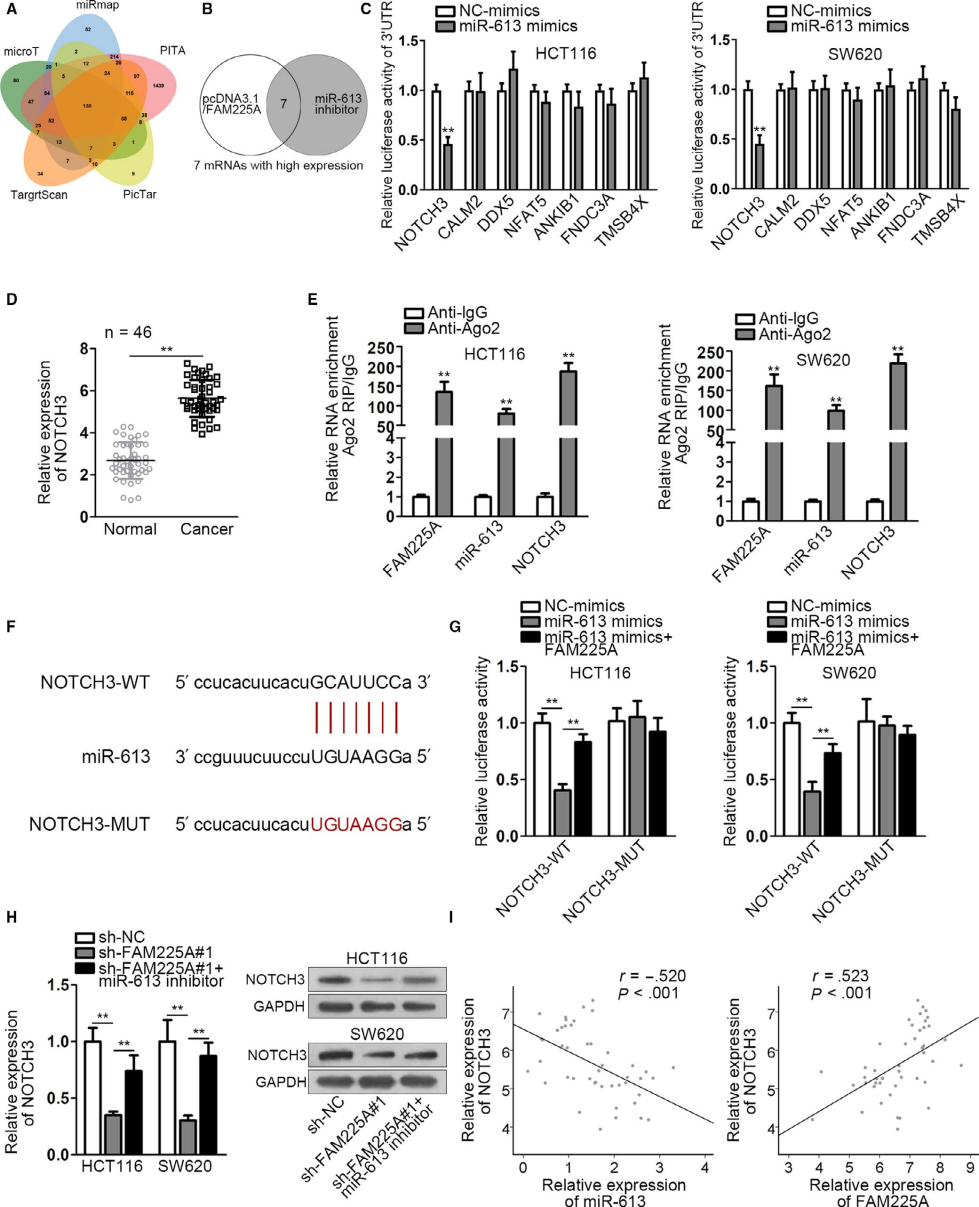
FAM225A modulates NOTCH3 expression by binding to miR‐613. A, Venn diagram was plotted to show the common mRNAs predicted by five bioinformatics tools (miRmap, TargetScan, microT, PITA, and PicTar). B, qRT‐PCR analysis of mRNA expressions. Relative qRT‐PCR results were provided in an additional file named as “Supplementary Table S1.” C, Applying luciferase reporter assay ascertained the target gene of miR‐613 among the candidate mRNAs. D, qRT‐PCR was adopted to assess NOTCH3 expression in cancer tissues and normal tissues. E. RIP assay verified that FAM225A, miR‐613, and NOTCH3 coexisted in RNA‐induced silencing complex (RISC). F, The binding sequences of miR‐613 and NOTCH3 were acquired from starBase website. G, Luciferase reporter assay was applied to attest the interaction relationship of miR‐613 and NOTCH3. H, NOTCH3 mRNA and protein levels were measured by qRT‐PCR and western blot analysis. I, Pearson correlation analysis of the relationship between miR‐613 and NOTCH3, as well as FAM225A and NOTCH3. All data were manifested as the mean ± SD. **P < .01, ***P < .001

Five bioinformatics tools (miRmap, TargetScan, microT, PITA, and PicTar) were utilized to predict the potential messenger‐RNAs (mRNAs) which could bind with miR‐613 (Figure 4A). Then, through overexpressing FAM225A and interfering miR‐613, we screened seven mRNAs with high expression (Figure 4B). The relative qRT‐PCR results were provided in an additional file named as “Supplementary Table S1.” Additionally, luciferase reporter assay was performed by co‐transfecting miR‐613‐WT/MUT and seven mRNAs into HCT116 and SW620 cells. The result suggested that only the luciferase activity of NOTCH3 group was declined by miR‐613 mimics (Figure 4C). In addition, NOTCH3 also has been reported to play an oncogene part in other cancers.^18^ Hence, NOTCH3 was selected as the target gene of miR‐613. qRT‐PCR analysis reflected the much lower NOTCH3 expression in normal tissues than in cancer tissues (Figure 4D). In addition, from the results of RIP assay, FAM225A, miR‐613, and NOTCH3 were all enriched in Ago2‐precipitated products, which implied that FAM225A, miR‐613, and NOTCH3 could coexist in RNA‐induced silencing complex (RISC) (Figure 4E). We acquired the binding sequence of NOTCH3 and miR‐613 from starBase website and mutated the potential binding sequence of NOTCH3 on miR‐613 (Figure 4F). Luciferase reporter rescue assay showed that the luciferase activity of NOTCH3‐WT was decreased by miR‐613 upregulation, while was recovered by overexpressing FAM225A. There was no change in NOTCH3‐MUT group (Figure 4G). Furthermore, we co‐transfected sh‐FAM225A#1 and miR‐613 inhibitor into HCT116 and SW620 cells; as the results of qRT‐PCR and western blot depicted, NOTCH3 expression and protein level were declined by FAM225A knockdown, while were upregulated by miR‐613 downregulation (Figure 4H). Furthermore, from Pearson correlation analysis, we uncovered the inverse correlation of NOTCH3 and miR‐613, as well as the positive correlation between NOTCH3 and FAM225A (Figure 4I). We concluded that FAM225A modulated NOTCH3 expression via binding with miR‐613.

### 3.5 FAM225A facilitates CRC progression via miR‐613/NOTCH3 axis

**FIGURE 5:**
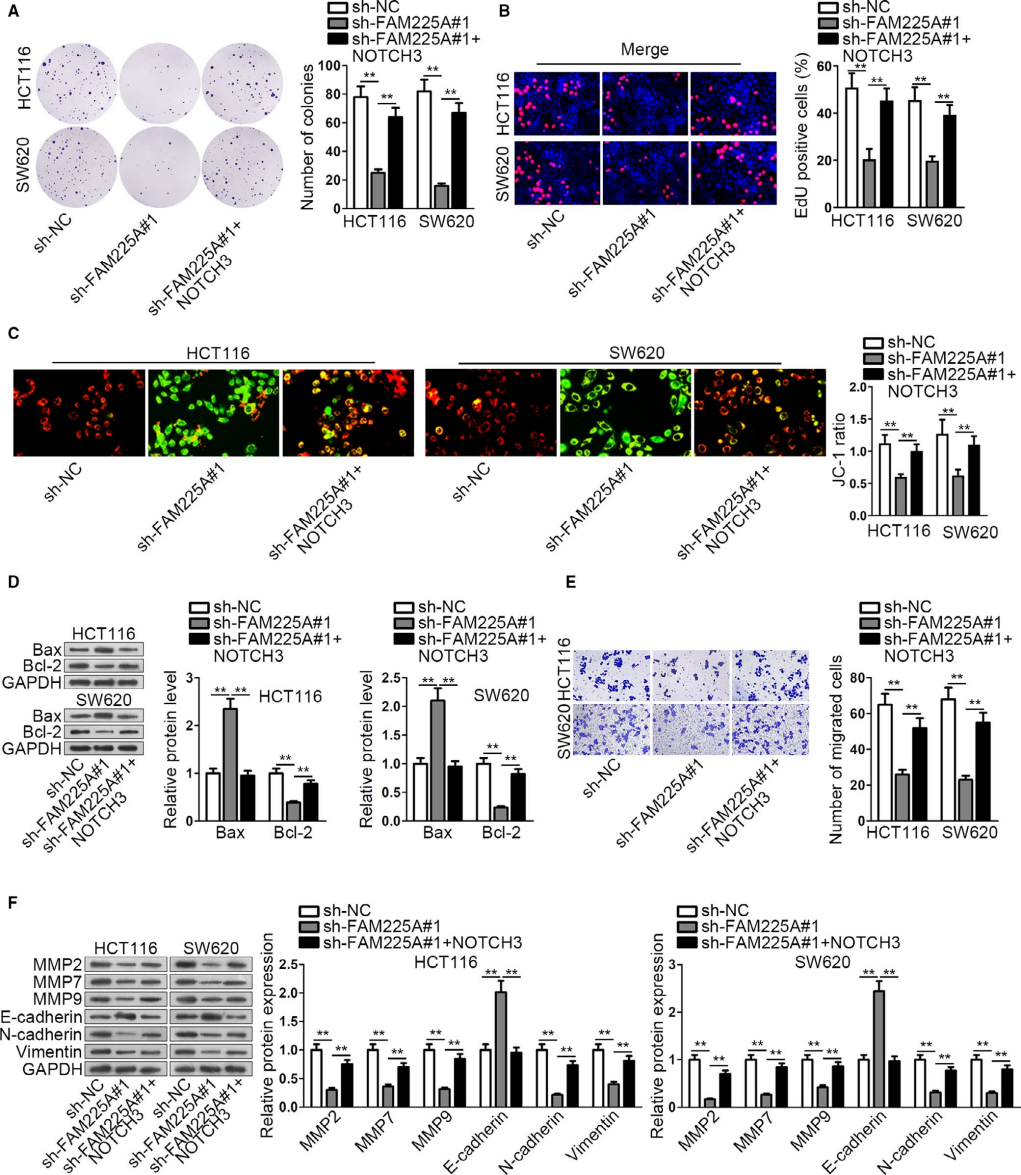
FAM225A facilitates CRC progression via miR‐613/NOTCH3 axis. A and B, HCT116 and SW620 cell lines were co‐transfected with sh‐FAM225A#1 and pcDNA3.1/NOTCH3. Colony formation assay and EdU assay were conducted to test cell proliferation capacity. C, JC‐1 assay was applied to test cell apoptosis ability. D, Apoptosis‐associated protein levels were measured by western blot assay. E, Transwell assay manifested cell migration capability. F, Migration‐related protein and EMT progression‐associated protein levels were determined via western blot. Results were presented as the mean ± SD. **P < .01

The functional rescue experiments were carried out to proof that FAM225A influenced CRC progression via modulating miR‐613/NOTCH3 axis. HCT116 and SW620 cell lines were co‐transfected with sh‐FAM225A#1 and pcDNA3.1/NOTCH3. From the results of colony formation assay and EdU assay, we concluded that cell proliferation capacity was impaired by FAM225A knockdown, while was restored by NOTCH3 increase (Figure 5A,B). By comparison, as JC‐1 assay and associated protein levels detection indicated, cell apoptosis capability was promoted by FAM225A downregulation, whereas was repressed with elevated NOTCH3 (Figure 5C,D). Meanwhile, cell migration capacity was restricted by FAM225A depletion, whereas was completely recovered via overexpressing NOTCH3 (Figure 5E). Furthermore, from the change of migration‐related protein and EMT progression‐correlated protein levels, we also found that FAM225A downregulation suppressed migration capacity and EMT progression, while augmenting NOTCH3 rescued them (Figure 5F). Moreover, the rescue assays were conducted again with silenced NOTCH3 to rescue overexpressed FAM225A. It was revealed that upregulated FAM225A promoted cell proliferation, migration, EMT process, and inhibited cell apoptosis. However, the effects of upregulated FAM225A was completely rescued by downregulation of NOTCH3 (Figure S3A‐H). Therefore, we drew a conclusion that FAM225A facilitated CRC progression via regulating miR‐613/NOTCH3 axis.

## 4 DISCUSSION

MiRNAs are regarded as the potential markers of auxiliary diagnosis, personalized treatments, and prognosis estimation.^19^ More than 1000 kinds of human miRNAs have been uncovered to change one third of human genes expression levels, and to control the alterations of cell functions.^20^, ^21^ As a member of miRNA family, miR‐613 exhibits characteristic advantages in diagnosis and treatments of multiple cancers, including non‐small cell lung cancer, bladder cancer, and ovarian cancer.^22^, ^23^, ^24^ For example, miR‐613 was reported to have obvious lower expression in bladder cancer cell lines than in normal cells. MiR‐613 exerts tumor‐suppressing effects by directly targeting SphK1 in bladder cancer.^23^ MiR‐613 repressed breast cancer cell proliferation and invasion via negatively modulating vascular endothelial growth factor^25^. Similarly, in our study, miR‐613 was found presenting lower expression in CRC tissues than normal tissues. Besides, as the downstream gene of FAM225A, miR‐613 could bind to FAM225A and inversely associated with it. On the other hand, as the upstream gene of NOTCH3, miR‐613 could also bind with NOTCH3 and negatively associated with it. FAM225A regulated NOTCH3 expression via competitively sponging miR‐613. MiR‐613 exerted important effects as a mediator between FAM225A and NOTCH3 in CRC cells. Hence, miR‐613 served as a tumor‐repressing gene, offering some references for searching new treatment targets against CRC.

NOTCH family participates in the occurrence and development of multiple cancers through modulating tumor microenvironment, tumor formation, progression, angiogenesis, migration, and invasion.^26^ NOTCH is also correlated with cancer cell metabolism, cell survival, drug resistance, as well as genomic instability. NOTCH4 has been discovered displaying much higher expression in liver metastases than in the normal mucosa. The upregulated NOTCH4 expression was closely associated with advanced stage of colorectal cancer.^27^ Interestingly, in this study, NOTCH3 was also verified having higher expression in CRC tissues than in normal tissues. Besides, NOTCH3 was the target gene of miR‐613, and was indirectly regulated by FAM225A in CRC cells. NOTCH3 was negatively correlated with miR‐613, whereas was positively associated with FAM225A. Therefore, we speculated that FAM225A may promote CRC progression through miR‐613/NOTCH3 axis. As predicted, from the rescue experiments, we draw the conclusion that FAM225A enhanced cell proliferation, migration, and EMT progress, while blocked cell apoptosis ability via positively modulating miR‐613/NOTCH3 axis. Therefore, FAM225A/miR‐613/NOTCH3 axis may play a tumor‐facilitating role in CRC cell progression. These data manifested the pivotal effect of FAM225A/miR‐613/NOTCH3 pathway in CRC cell proliferation, apoptosis, and migration process. It may also provide some theoretical basis and different perspective for identifying new biomarkers against CRC.

To draw a conclusion, FAM225A facilitated CRC occurrence and development through competitively binding with miR‐613 and positively regulating NOTCH3 expression.

## CONFLICT OF INTEREST

None.

## AUTHOR CONTRIBUTIONS

Xuexiu Zhang conceived and designed the study. Xuexiu Zhang, Haoling Shi, Jianning Yao, and Yanle Li performed the experiments. Bing Gao, Yanzhen Zhang, and Chunfeng Wang assisted with analyzing the data. Xuexiu Zhang drafted the manuscript, which was reviewed by Haining Zhou and Lianfeng Zhang.

## ETHICAL STATEMENT

Participants signed written informed consent. And this study was approved by the Ethics committee of the First Affiliated Hospital of Zhengzhou University.

## Supporting information

Fig S1

Fig S2

Fig S3

Table S1

## Data Availability

Research data are not shared.
