# Supplementary figures and images for: FAM225A facilitates colorectal cancer progression by sponging miR‐613 to regulate NOTCH3
Source: Cancer Med. 2020 Apr 28;9(12):4339–49. doi: 10.1002/cam4.3053 (PMC7300395; doi:10.1002/cam4.3053)

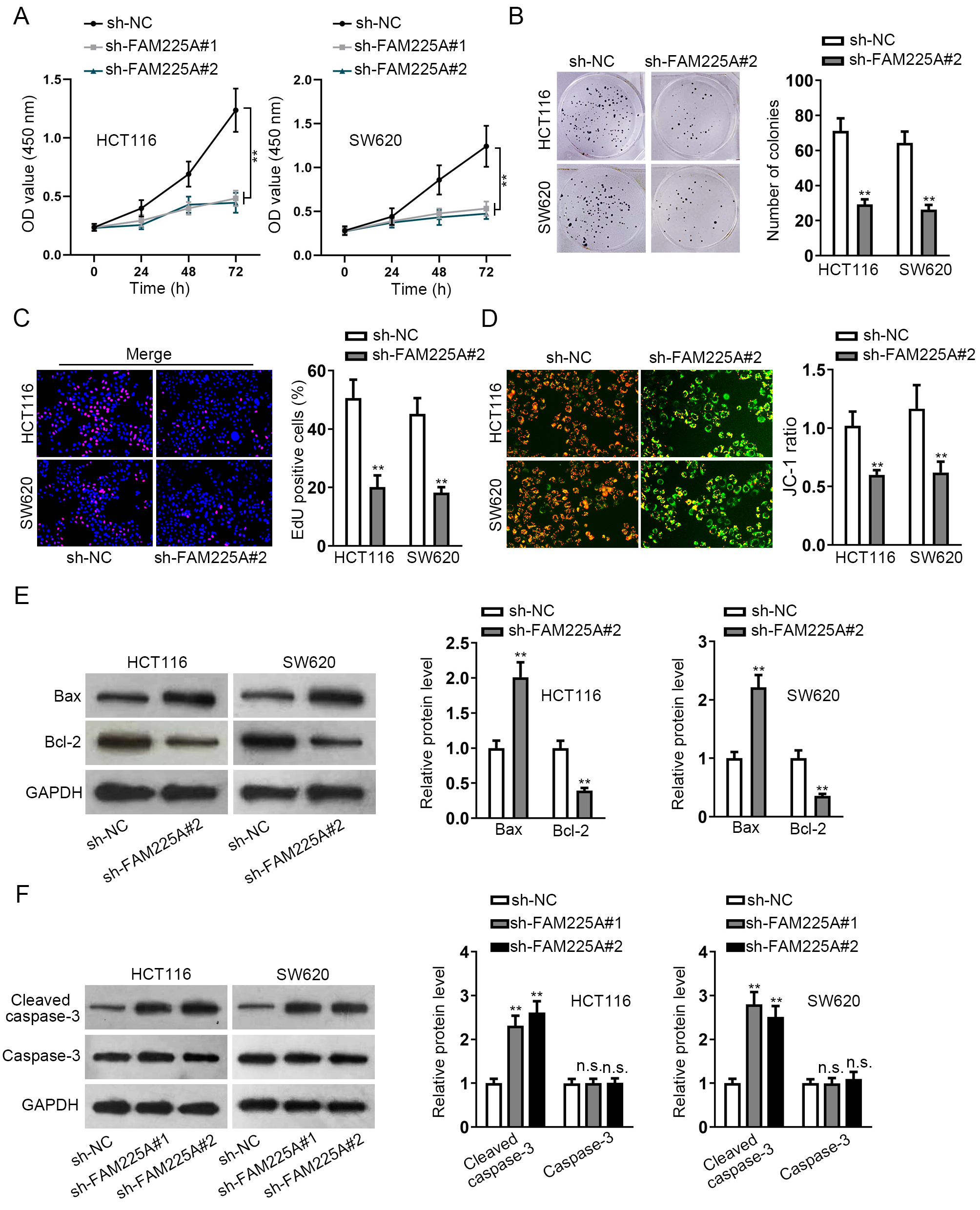

Supplement: Supplementary file 1 — Fig S1 [file CAM4-9-4339-s001.tif]

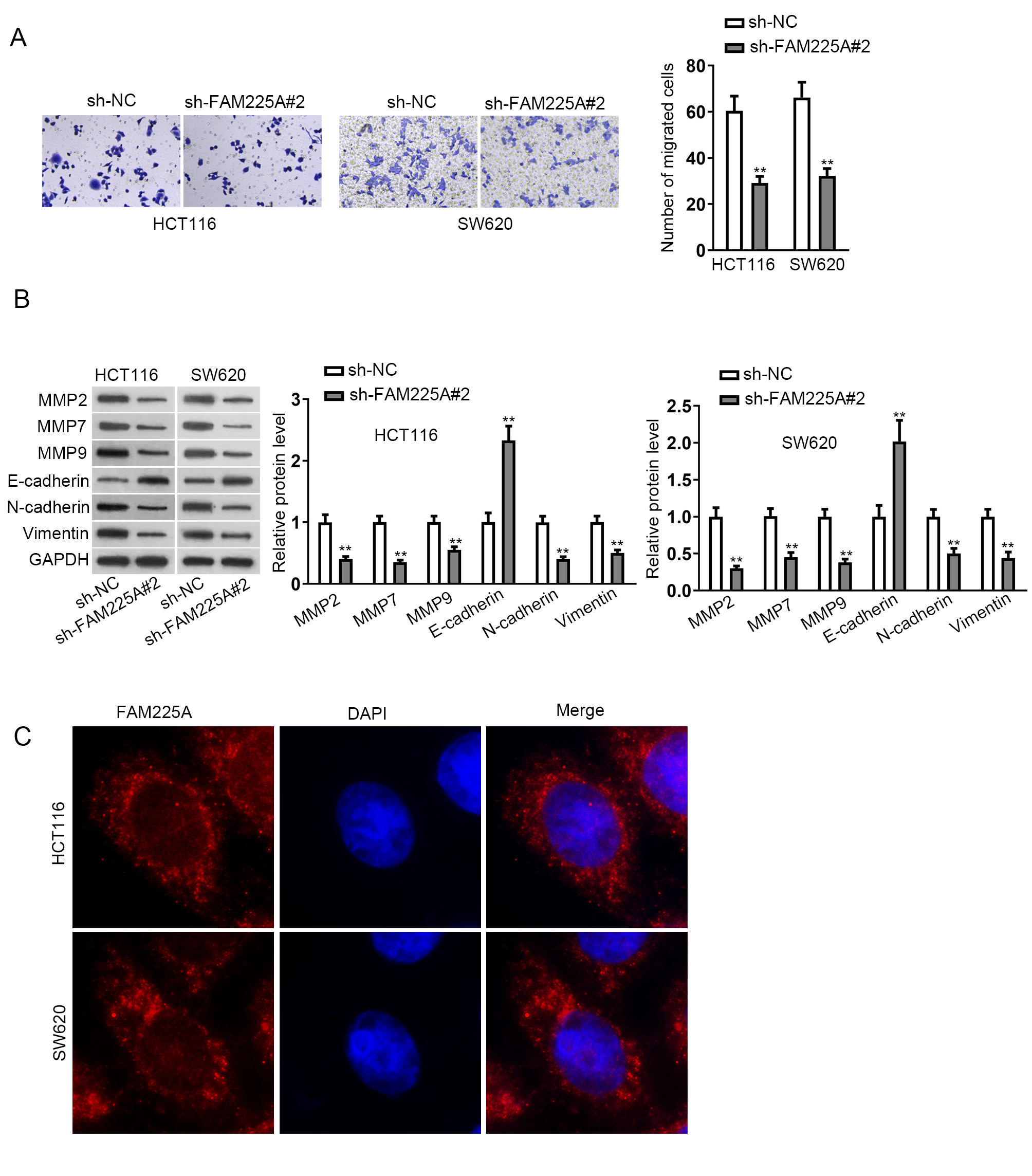

Supplement: Supplementary file 2 — Fig S2 [file CAM4-9-4339-s002.tif]

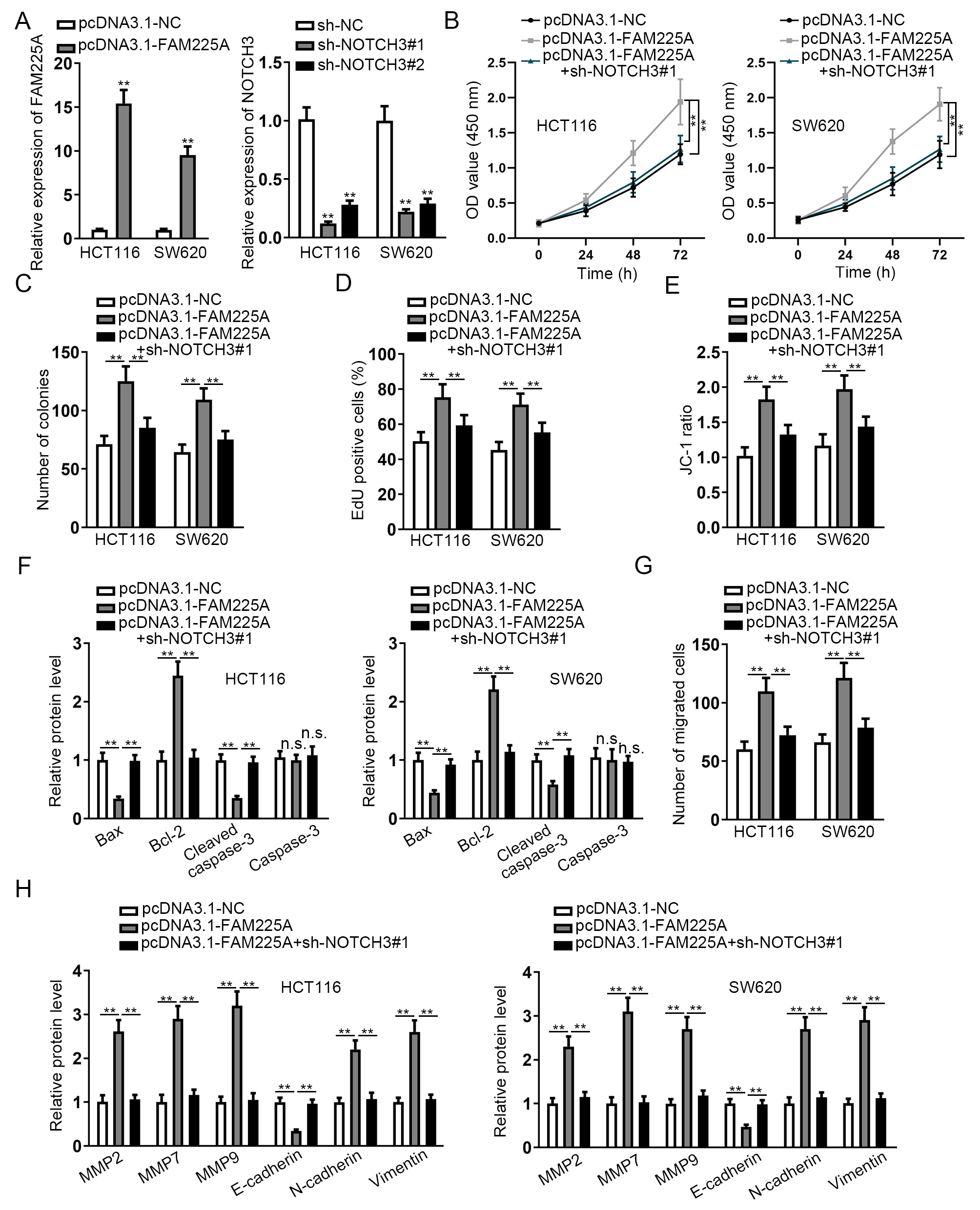

Supplement: Supplementary file 3 — Fig S3 [file CAM4-9-4339-s003.tif]
